# Supplementary material for: Strategic Planning of a Joint SARS-CoV-2 and Influenza Vaccination Campaign in the UK
Source: Vaccines (Basel). 2024 Feb 3;12(2):158. doi: 10.3390/vaccines12020158 (PMC10891881; doi:10.3390/vaccines12020158)
Supplement: Supplementary file 1 [file vaccines-12-00158-s001.zip › vaccines-2831081-supplementary.pdf]

Strategic planning of joint SARS-CoV-2 and influenza vaccination campaign in the UK

Dauda Ibrahim [1,], Zoltán Kis [1,2], Maria M. Papathanasiou [1], Cleo Kontoravdi [1], Benoît Chachuat [1] and Nilay Shah [1]*

[1]The Sargent Centre for Process Systems Engineering, Department of Chemical Engineering, Imperial College London, London SW7 2AZ, UK; z.kis10@imperial.ac.uk (Z.K.); maria.papathanasiou11@imperial.ac.uk (M.M.P.); cleo.kontoravdi98@imperial.ac.uk (C.K.); b.chachuat@imperial.ac.uk (B.C.); n.shah@imperial.ac.uk (N.S.)

[2]Department of Chemical and Biological Engineering, The University of Sheffield, Sheffield S1 3JD, UK

*Correspondence: d.ibrahim@imperial.ac.uk

Contents

S1 Model input data 2

S1.1 Target population for COVID-19 booster 2

S1.2 Target population for flu vaccination 2

S1.3 Vaccine candidates recommended for COVID-19 booster and flu vaccination campaigns 3

S1.4 Vaccine storage technology 4

S1.5 Transport mode 4

S2 Structure of SARS CoV2 and influenza vaccine supply chain 4

S3 Mathematical formulation of SARS CoV2 and influenza vaccine supply chain 8

S3.2. Objective function 8

S3.3. Model constraints 10

S2.3.1. Material balance 10

S3.3.2. Tracking vaccine shippers: warehouses–regional store–clinics–warehouses 11

S3.3.3. Shelf life of vaccines at administration points 11

S3.3.4. Safety stock, maximum inventory, and bounds on QC checks, production rate, and import rate. 12

S3.3.5. Number of trips 13

S3.3.6. SARS CoV2 and influenza vaccines demand 14

S2.3.7. Non-negativity constraints 14

S3.4. Network configuration constraints 14

S1 Model input data

S1.1 Target population for SARS-CoV-2 vaccine

Table S1 Target population for SARS-CoV-2 vaccine

Cohort	Priority	Population	Vaccine
Care home residents	1	354628	Pfizer/Moderna
Residential Care workers	1	505622	Pfizer/Moderna
65+	2	11679797	Pfizer/Moderna
Healthcare workers	3	2333818	Pfizer/Moderna
Social care workers	3	1400155	Pfizer/Moderna
At risk (under 65)	4	7503400	Pfizer/Moderna
50-64	5	7552350	Pfizer/Moderna
Pregnant women	6	719011	Pfizer/Moderna

S1.2 Target population for influenza vaccination

Table S2 Target population for influenza vaccination

Cohort	Priority	Population	Vaccines
Care home residents	1	354628	aQIV
Residential Care workers	1	505622	QIVc
65+	2	11679797	aQIV
Healthcare workers	3	2333818	QIVc
Social care workers	3	1400155	QIVc
At risk (under 65)	4	7488018	QIVc/QIVr
50-64	5	7552350	QIVc/QIVr
Pregnant women	6	719011	QIVc/QIVr
Aged 2	7	745449	LAIV
Aged 3	8	771730	LAIV
Reception (age 4-5)			LAIV
Year 1 (age 5-6 yrs)	9	1549865	LAIV
Year 2 (age 6-7 yrs)			LAIV
Year 3 (age 7-8 yrs)	10	1589828	LAIV
Year 4 (age 8-9 yrs)			LAIV
Year 5 (age 9-10 yrs)	11	1651751	LAIV
Year 6 (age 10-11 yrs)			LAIV
Year 7 (age 11-12 yrs)	12	1616686	LAIV
Under Aged 2		15382	QIVe

S1.3 Vaccine candidates recommended for SARS-CoV-2 and influenza vaccination campaigns

Table S3 Vaccine candidates recommended for SARS-CoV-2 and influenza vaccination campaigns

Item	Unit	Vaccine type-1	Vaccine type-2	Vaccine type-3	Vaccine type-4	Vaccine type-5	Vaccine type-6	Vaccine type-7
Name	[-]	Cominarty	Spikevax	Influvac Tetra	Flucelvax Tetra	VaxigripTetra	Fluenz Tetra	Fluad
Abbreviation	[-]	BNT162b2	mRNA-1234	QIVe	QIVc	QIVe or QIVr	LAIV	aQIV
Manufacturer	[-]	Pfizer-BioNTec	Moderna	Mylan	Seqirus	Sanofi Pasteur	AstraZeneca	Seqirus
Method of administration	[-]	Intramuscular	Intramuscular	Intramuscular	Intramuscular	Intramuscular	Nostril	Intramuscular
Vaccine presentation	[-]	Liquid	Liquid	Liquid	Liquid	Liquid	Nasal spray	Liquid
Dose(s) per vial	dose/vial	5	10	1	1	1	1	1
Storage temperature and shelf life	oC	. -90 to -60 (9 month)	2-8 (30 days)	2 to 8 (12 months)	2 to 8 (12 months)	2 to 8 (12 months)	2 to 8 (18 weeks)	2 to 8 (12 months)
		2-8 (5 days)	35-40 (up 12 hours)					
		35-40 (2 hr - up to 6 hr after dilution)	-20 (up to 6 months)					
Dose per patient	dose/patient	2	2	1	1	1	1	1
Schedule/days apart	day	21	28	N/A	N/A	N/A	N/A	N/A
Vaccine weight	g/dose	0.8522	0.8522	0.8522	0.8522	0.8522	0.8522	0.8522
Secondary packaging	vial/container	195	10	1	10	1	10	10
Vaccine cost per vial	$/dose	18.66	34.84	9.95	12.37	9.95	22.4	16.8

S1.4 Vaccine storage technology

Table S4 Maximum capacities of storage technology at central and regional warehouses as well as administration points

Storage device	Administration point	Regional stores	Warehouses	Unit
ULT freezer	30000	90000	150000	L
Freezer	30000	90000	150000	L
Fridge	30000	90000	150000	L

Table S5 Installation and operating cost of each storage technology

Storage device	Operating cost	Installed capital cost	Unit
ULT freezer	0.108	0.1158	USD/L
Freezer	0.1066	0.1048	USD/L
Fridge	0.0462	0.0719	USD/L

S1.5 Transport mode

Table S6 Unit transport cost and capacities of each transport mode

	Unit transport cost			
	Van	Truck	Airplane	Unit
	0.101849	0.257099	187.6063	USD/km
	Maximum storage capacities			
Vaccine type	Van	Truck	Airplane	Unit
Cominarty	421121	2601655	24642103	dose/trip
Spikevax	1801602	11130174	24642103	dose/trip
Influvac Tetra	107100	661653	24642103	dose/trip
Flucelvax Tetra	134476	830782	24642103	dose/trip
VaxigripTetra	107100	661653	24642103	dose/trip
Fluenz Tetra	105194	649878	24642103	dose/trip
Fluad	134476	830782	24642103	dose/trip

S2 Structure of SARS CoV2 and influenza vaccine supply chain

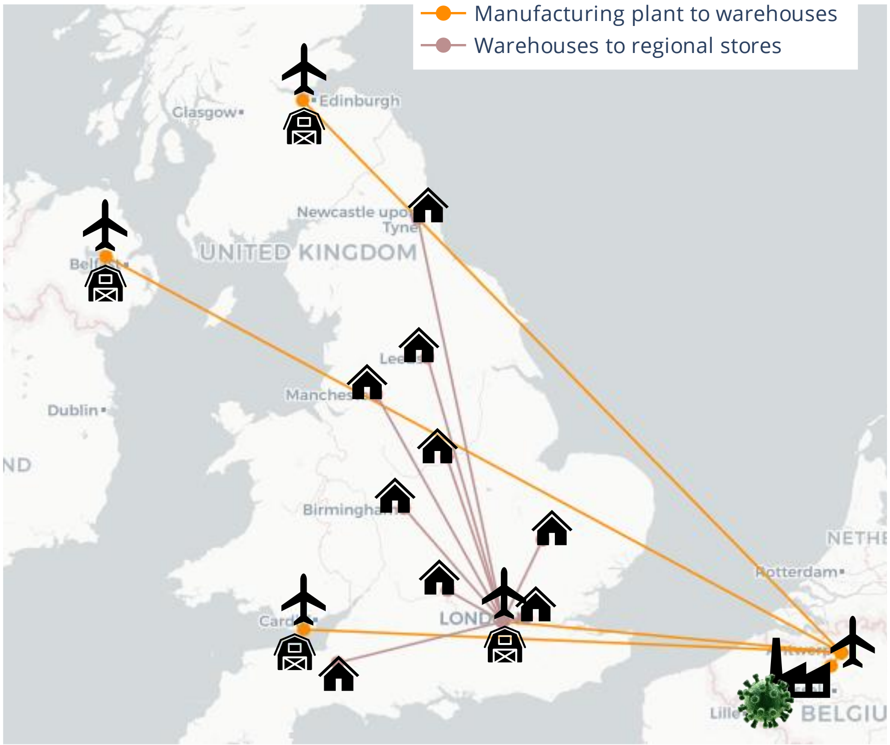

Figure S1. Structure of supply chain for Cominarty (BNT162b2) SARS CoV2 vaccine. The manufacturing plant is located in Puurs, Belgium while warehouses and regional stores are located across the UK: North east, North West, Yorkshire and The Humber, East Midlands, West Midlands, East of England, London, South East, South West, Wales, Scotland, Northern Ireland.

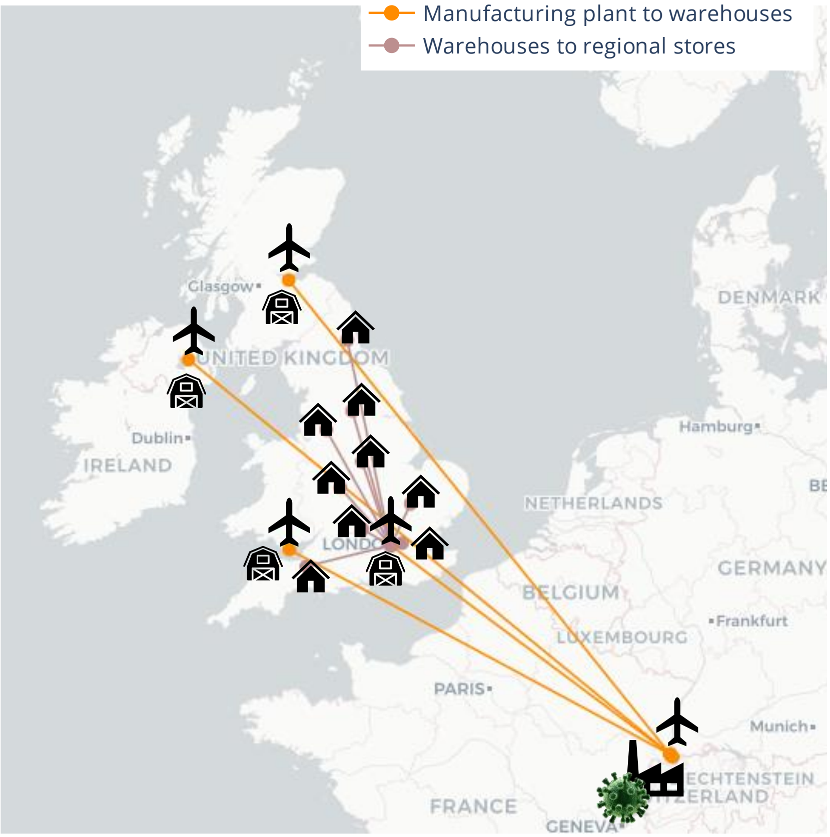

Figure S2. Structure of supply chain for Spikevax (mRNA-1234) SARS CoV2 vaccine. The manufacturing plant is located in Basel, Switzerland while warehouses and regional stores are located across the UK: North east, North West, Yorkshire and The Humber, East Midlands, West Midlands, East of England, London, South East, South West, Wales, Scotland, Northern Ireland.

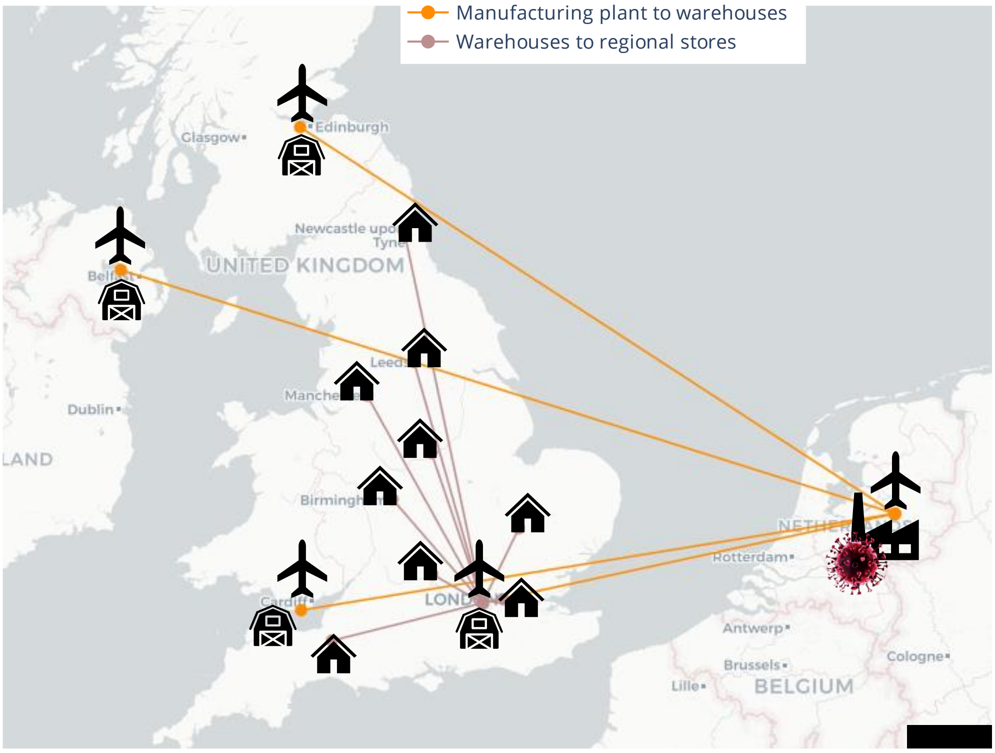

Figure S3. Structure of supply chain for Influvac Tetra (QIVe) influenza vaccine. The manufacturing plant is located in Olst, The Netherlands while warehouses and regional stores are located across the UK: North east, North West, Yorkshire and The Humber, East Midlands, West Midlands, East of England, London, South East, South West, Wales, Scotland, Northern Ireland.

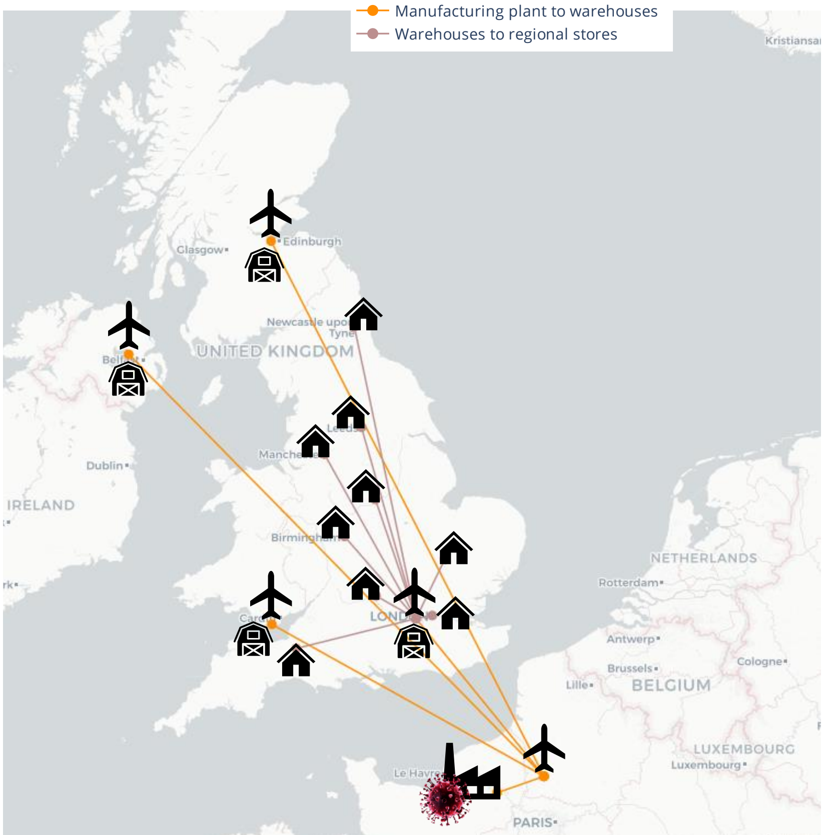

Figure S4. Structure of supply chain for Vaxigrip Tetra (QIVe or QIVr) influenza vaccine. The manufacturing plant is located in Val-de-Reuil, France while warehouses and regional stores are located across the UK: North east, North West, Yorkshire and The Humber, East Midlands, West Midlands, East of England, London, South East, South West, Wales, Scotland, Northern Ireland.

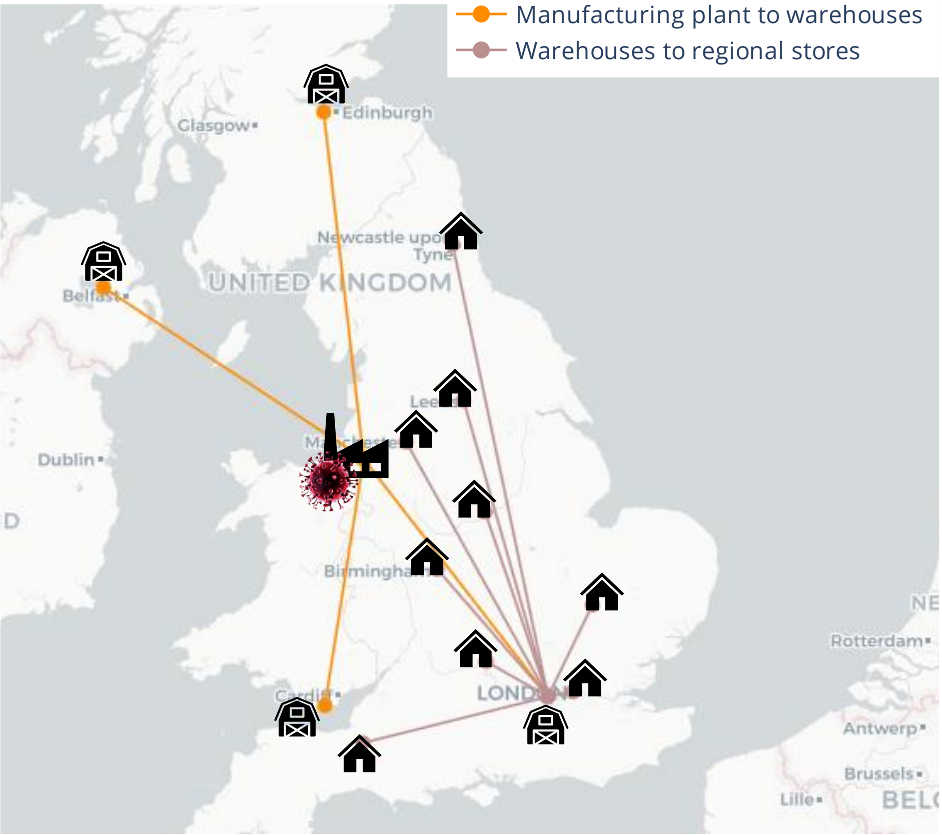

Figure S5. Structure of supply chain for influenza vaccine candidates: Flucelvax Tetra (QIVc) and Fluad (aQIV) produced by Seqirus and Fluenz Tetra (LAIV) produced by AstraZeneca. The manufacturing plant is located in Liverpool, UK while warehouses and regional stores are located across the UK: North east, North West, Yorkshire and The Humber, East Midlands, West Midlands, East of England, London, South East, South West, Wales, Scotland, Northern Ireland.

S3 Mathematical formulation of SARS CoV2 and influenza vaccine supply chain

S3.2. Objective function

The objective function considered in this work is total cost (TC) incurred over the entire vaccination period and is defined as follows.

$$\mathrm{TC} = \mathrm{MT} + (\mathrm{CT} + \mathrm{OT} + \mathrm{TT}) \cdot \mathrm{VT} \tag{S1}$$

where CT is the annualised installed capital cost of supply chain equipment and building, OT is the total cost of operating the equipment and building throughout the vaccination period, TT is the transportation cost, MT denotes miscellaneous cost, and VT denotes vaccination time frame. The miscellaneous cost includes the cost of administering vaccines to target patients, vaccine procurement, quality control checks, thermal shippers, and dry ice.

The annualised installed capital cost, CT, is the sum of installed cost of supply chain assets, including storage devices (fridge, freezer, and ultra-low temperature freezer) at central and regional warehouses and building infrastructure.

$$CT = \sum_{vst, w \in W} V2_{vwt}^{max} \cdot C1_{sw} + \sum_{vst, r \in R} V3_{vrt}^{max} \cdot C2_{sr} \tag{S2}$$

where $V2_{vwt}^{max}$ and $V3_{vrt}^{max}$ are prevailing capacities and/or inventory of various vaccine candidates at central warehouses and regional stores; $C1_{sw}$ and $C2_{sr}$ are unit installed capital cost, denoting the installation cost of central warehouses and regional stores respectively. The unit installed capital cost takes into account building cost and cost of cold chain devices.

The operating cost, OT, is define as the vaccine inventory at a given time period multiply by unit inventory holding cost.

$$OT = \sum_{wt} V2_{vwt}^{max} \cdot O1_{sw} + \sum_{rt} V3_{vrt}^{max} \cdot O2_{sr} \tag{S3}$$

where $O1_{sw}$ and $O2_{sr}$ are unit inventory holding cost at central warehouses and regional stores respectively. The unit inventory holding cost takes into account for building utilities cost (electricity), labour cost, and cost of operating cold chain devices.

Equation S4 calculates the total transportation cost, TT, between supply chain entities, which is define as the unit transportation cost for a specific transport mode multiply by the number of trips covered at a given time period. The unit transportation cost accounts for travel distance, driver wages, fuel and vehicle maintenance cost, and annualised capital cost of transport vehicle.

$$\begin{aligned} TT = & \sum_{vmwjt} U1_{mwj} \cdot N1_{vmwjt} + \sum_{viwjt} U2_{iwj} \cdot N2_{viwjt} + \sum_{vwrjt} U3_{wrj} \cdot N3_{vwrjt} \\ & + \sum_{vrcjt} U4_{rcj} \cdot N4_{vrcjt} + \sum_{vcwjt} U5_{cwj} \cdot N5_{vcwjt} \end{aligned} \tag{S4}$$

where U and N denote, respectively, unit transportation cost and number of trips between supply chain entities.

As mentioned earlier, the miscellaneous cost, MT, comprise of cost of administering vaccines to target patients, vaccine procurement, quality control checks, thermal shippers, and dry ice.

$$MT = C_S + C_D + C_A^a + C_A^b + C_V + C_Q \tag{S5}$$

Equations S6 and S7 estimate the cost thermal shippers, C_S, and dry ice, C_D, respectively. The cost of dry ice can be calculated as the total dry ice required multiplied by the unit price of dry ice (see Equation S7); each full shipper leaving warehouses is loaded with sufficient dry ice to maintain the sub-zero storage temperature of -80°C.

The cost of vaccine shippers can be estimated by multiplying the number of shippers supplied at the start of the vaccination campaign by the unit price of shipper (see Equation S6). Here, it is assumed that no shipper is damage and/or replace throughout the vaccination campaign.

$$C_S = \sum_{vwt} NTS_{vwt}^{W} \cdot UPS \tag{S6}$$

$$C_D = \sum_{vwrjt} \frac{Q3_{vwrjt}}{DPS} \cdot DIS \cdot UPD \tag{S7}$$

where NTS_{vwt}^{W} is the total number of thermal shippers at warehouses during the entire vaccination period, considering re-usage of thermal shippers, that is, shippers are recycled back to their corresponding warehouses once vaccines are unloaded at vaccine administration points. UPS, DPS, DIS, and UPD denote, respectively, unit price of shipper, dose of vaccine per shipper, dry ice required per shipper, and unit price of dry ice.

Equations S8 and S9 estimate the cost of administering influenza vaccines, C_A^a, and SARS CoV2 vaccines, C_A^b, respectively.

$$C_A^a = \sum_{v \in FLU, ct} D_{vct}^{S} \cdot UPV^{a} \tag{S8}$$

$$C_A^b = \sum_{v \in COV, ct=3} D_{vct}^{S} \cdot UPV^{b} \tag{S9}$$

The total doses of vaccines (influenza and SARS-CoV-2) supplied and administered to patients at vaccine administration points is denoted by D_{vct}^{S}, while UPV^{a} and UPV^{b} represent the cost of administering a single dose of influenza and SARS-CoV-2 vaccines respectively.

The total cost of procuring,C_V, both influenza and SARS-CoV-2 vaccines is estimated by Equation S10, where PPD_v represents the price per dose of vaccines.

$$C_V = \sum_{vct} D^S_{vct} \cdot PPD_v \tag{S10}$$

$$C_Q = \sum_{vwt} F3_{vwt} \cdot UPQ \tag{S11}$$

Lastly the cost of quality control checks, C_Q, is defined as the quantity of vaccines, $F3_{vwt}$, that undergo quality control checks at warehouse multiply by the unit price of UPQ, see Equation S11.

S3.3. Model constraints

S3.3.1. Material balance

The flow of vaccines to and from each entity of the vaccine supply is defined by the material balance constraints. As shown in **Figure 1**, the entities of the supply chain include manufacturing plants, warehouses, regional stores, and administration points. In Equation S12, $I1_{vmt}$ denotes the inventory of vaccines v in manufacturing plant m at time period t, which is calculated as the quantity of vaccine v produced at manufacturing plant m ($F1_{vmt} = P_{vmt}$) at time t minus the quantity of vaccine leaving the plant at time t ($F2_{vmt} = \sum_{mj} Q1_{vmwjt}$) plus the inventory of vaccines in the previous time period ($I1_{mt-1}$).

$$I1_{vmt} = F1_{vmt} - F2_{vmt} + I1_{vmt-1} \qquad \forall\, v \in V, m \in M, t \in T \tag{S12}$$

$$I2in_{vwt} = F3_{vwt} - QC_{vwt} + I2inv_{wt-1} \qquad \forall\, v \in V, w \in W, t \in T \tag{S13}$$

$$I2out_{vwt} = QC_{vwt-2} - F4_{vwt} + I2out_{vwt-1} \qquad \forall\, v \in V, w \in W, t \in \mathrm{T} \tag{S14}$$

$$I3_{vrt} = F5_{vrt} - F6_{vrt} + I3_{vrt-1} \qquad \forall\, v \in V, r \in R, t \in T \tag{S15}$$

$$I4_{vct} = F7_{vct} - F8_{vct} - W^C_{vct} + I4_{vct-1} \qquad \forall\, v \in V, c \in C, t \in T \tag{S16}$$

Similarly, Equations S14 to S16 are used to estimate the inventory of vaccines in warehouses, regional stores, and administration points, where $F3_{vwt} = \sum_{mj} Q1_{vmwjt} + \sum_{ij} Q2_{viwjt}$; $F4_{vwt} = \sum_{rj} Q3_{vwrjt}$; $F5_{vrt} = \sum_{wj} Q3_{vwrjt}$; $F6_{vrt} = \sum_{cj} Q4_{vrcjt}$; $F7_{vct} = \sum_{rj} Q4_{vrcjt}$; and $F8_{vct} = D^S_{vct}$;

In warehouses, vaccines are expected to undergo quality control (QC) checks, which can take up to two weeks. To account for the delay due to QC, the inventory of vaccines at warehouse is partitioned into $I3inv_{wt}$ and $I3out_{vwt}$, denoting vaccine inventory before and after QC respectively. The inventory of vaccines before QC is calculated by subtracting the quantity of vaccines leaving for QC checks from the sum of vaccines arriving from fill-finish plants, oversees imports, and vaccine inventory at previous time period. The inventory of vaccines after QC is calculated by subtracting the quantity of vaccines leaving warehouses from vaccines that have undergone QC checks and vaccine inventory at previous time period. The negative two subscript indicates that only vaccines that have been held for two weeks are allowed to leave warehouses. Here, each time period is equivalent to one week.

S3.3.2. Tracking vaccine shippers: warehouses–regional store–clinics–warehouses

In addition to vaccines, the supply chain also manages the flow on thermal shippers used in transporting vaccine candidates that require ultra-low temperature cooling. In this supply chain, the thermal shippers flow from warehouses to administration points via regional stores, they are returned back to their corresponding warehouses once vaccines are unloaded at administration points.

Equations S17, S19, and S20 are used to calculate the number of full shippers in warehouses (NF^{W}_{vwt}), regional stores (NF^{R}_{vrt}), and administration points (NF^{C}_{vct}), whereas Equations S18 and S21 calculate the number of empty shippers (NE^{W}_{vwt} and NE^{C}_{vct}). The number of full shippers in warehouses, regional stores, and administration points is equal to shippers arriving plus shippers from pervious time period less shippers leaving the facilities. Likewise, the number of empty shippers is equal to empty shippers arriving plus empty shippers from previous time period less the number of empty shippers loaded with vaccines ready to be shipped from warehouses.

$$NF^{W}_{vwt} = QC_{vwt-2}/DPS - F4_{vwt}/DPS + NF^{W}_{vwt-1} \qquad \forall\, v \in V, w \in W, t \in T \qquad \text{(S17)}$$

$$NE^{W}_{vwt} = \textstyle\sum_{cj} Q5_{vcwjt}/DPS - F4_{vwt}/DPS + NE^{W}_{vwt-1} \qquad \forall\, v \in V, w \in W, t \in T \qquad \text{(S18)}$$

$$NF^{R}_{vrt} = F5_{vrt}/DPS - F6_{vrt}/DPS + NF^{R}_{vrt-1} \qquad \forall\, v \in V, r \in R, t \in T \qquad \text{(S19)}$$

$$NF^{C}_{vct} = F7_{vct}/DPS - F8_{vct}/DPS - W^{C}_{vct}/DPS + NF^{C}_{vct-1} \qquad \forall\, v \in V, c \in C, t \in T \qquad \text{(S20)}$$

$$NE^{C}_{vct} = F8_{vct}/DPS - \textstyle\sum_{wj} Q5_{vcwjt}/DPS + W^{C}_{vct}/DPS + NE^{C}_{vct-1} \quad \forall\, v \in V, c \in C, t \in T \qquad \text{(S21)}$$

Equation S22 calculates the quantity of thermal shippers returned back to warehouses from administration points. The term W^{C}_{vct} denotes vaccine wastage at administration point.

$$\sum_{wj} Q5_{vcwjt} = F8_{vct}/DPS + W^{C}_{vct}/DPS \qquad \forall\, v \in V, c \in C, t \in T \qquad \text{(S22)}$$

S3.3.3. Shelf life of vaccines at administration points

All vaccines have a corresponding shelf life after which the drug substance become ineffective and may cause significant side effects. With the exception of the BNT162b2 SARS-CoV-2 vaccine considered in the work, all other vaccine candidates have shelf lives longer than the vaccination timeframe of seven months, which is the timeframe recommended by the UK government [49–51]. BNT162b2 has a shelf life of one week when stored inside a thermal shipper and requires a sufficient quantity of dry ice to keep the temperature at -80°C. To avoid using expired vaccines at administration points, Equation S23 is included in the model to ensure that vaccines requiring ULT cooling stay at administration points for one week only. Any unused vaccines are discarded as waste after one week.

$$NF^{C}_{vct} \leq \sum_{vt}^{t+1} D^{S}_{vct} \Big/ DPS \qquad \forall\, c \in C, t \in T, v \in ULT \qquad \text{(S23)}$$

S3.3.4. Safety stock, maximum inventory, and bounds on QC checks, production rate, and import rate.

Safety stocks are used to complement vaccine supply, especially when there is a supply shortage during a vaccination campaign. This work assumes that safety stocks are stored at central warehouses only. Equation S24 defines the safety that should be maintained at central warehouses, which corresponds to the weekly average vaccine demand (AWD_{vw}) at administration points served by the warehouses.

$$I2in_{vwt} \geq AWD_{vw} \cdot E2_w \qquad \forall\, v \in V, w \in W, t \in T \qquad \text{(S24)}$$

The term $E2_w$ is a binary variable that takes a value of one if warehouse w is installed and zero otherwise.

Equations S25 to S28 defines the maximum inventory of vaccines that can be stored at each supply chain entity.

$$\sum_{v \in ST(s)} I1_{vmt} \cdot \varphi_v \leq I1_{sm}^{max} \cdot E1_m \qquad \forall\, s \in S, m \in M, t \in T \qquad \text{(S25)}$$

$$\sum_{v \in ST(s)} (I2in_{vwt} + I2out_{vwt}) \cdot \varphi_v \leq I2_{sw}^{max} \cdot E2_w \qquad \forall\, s \in S, w \in S, t \in T \qquad \text{(S26)}$$

$$\sum_{v \in ST(s)} I3_{vrt} \cdot \varphi_v \leq I3_{sr}^{max} \cdot E3_r \qquad \forall\, s \in S, r \in R, t \in T \qquad \text{(S27)}$$

$$\sum_{v \in ST(s)} I4_{vct} \cdot \varphi_v \leq I4_{sc}^{max} \cdot E4_c \qquad \forall\, s \in S, c \in C, t \in T \qquad \text{(S28)}$$

where $ST(s)$ denotes the set of vaccines v that can be stored in storage technology s; φ_v represents vaccines litre per dose; $I1_{sm}^{max}$, $I2_{sw}^{max}$, $I3_{sr}^{max}$, and $I4_{sc}^{max}$ represent maximum capacity of storage technologies at different levels of the supply chain; $E1_m$, $E2_w$, $E3_r$, and $E4_c$ takes a value of one if manufacturing plants, central warehouses, regional stores, and administration points are installed and zero otherwise.

$$QC_{vwt} \leq QC_{vw}^{max} \qquad \forall\, v \in V, w \in W, t \in T \qquad \text{(S29)}$$

$$M_{vit} \leq I_{vi}^{max} \qquad \forall\, v \in V, i \in I, t \in T \qquad \text{(S30)}$$

$$P_{vmt} \leq P_{vm}^{max} \qquad \forall\, v \in V, m \in M, t \in T \qquad \text{(S31)}$$

Equations S29 to S31 defines the maximum QC capacity at warehouses, quantity of vaccines imported from oversees, and production rate at manufacturing plants respectively.

$$I1_{vmt} \leq V1_{vmt}^{max} \qquad \forall\, v \in V, m \in M, t \in T \qquad \text{(S32)}$$

$$I2in_{vwt} + I2out_{vwt} \leq V2_{vwt}^{max} \qquad \forall\, v \in V, w \in W, t \in T \qquad \text{(S33)}$$

$$I3_{vrt} \leq V3_{vrt}^{max} \qquad \forall\, v \in V, r \in R, t \in T \qquad \text{(S34)}$$

$$I4_{vct} \leq V4_{vct}^{max} \qquad \forall\, v \in V, c \in C, t \in T \qquad \text{(S35)}$$

In Equations S32 to S35, $V1_{vmt}^{max}$, $V2_{vwt}^{max}$, $V3_{vrt}^{max}$, and $V4_{vct}^{max}$ denote the prevailing storage capacity needed/inventories of vaccines at manufacturing plants, central warehouses, regional stores, and administration points.

S3.3.5. Number of trips

Equations S36 to S40 calculate the number of trips between supply chain entities. Here $N1_{vmwjt}$, $N2_{viwjt}$, $N3_{vwrjt}$, $N4_{vrcjt}$, and $N5_{vcwjt}$ denote the number of trips between manufacturing plant and central warehouses, imports to central warehouses, central warehouses to regional stores, regional stores to clinics, and clinics to warehouses. Also, Q_{vj}^{max} denotes the quantity of vaccine that can be shipped transport mode j per trip.

$$N1_{vmwjt} = 2 \cdot Q1_{vmwjt}/Q_{vj}^{max} \qquad \forall\, v \in V, m \in M, w \in W, j \in J, t \in T \qquad \text{(S36)}$$

$$N2_{viwjt} = 2 \cdot Q2_{viwjt}/Q_{vj}^{max} \qquad \forall\, v \in V, i \in I, w \in W, j \in J, t \in T \qquad \text{(S37)}$$

$$N3_{vwrjt} = 2 \cdot Q3_{vwrjt}/Q_{vj}^{max} \qquad \forall\, v \in V, w \in W, r \in R, j \in J, t \in T \qquad \text{(S38)}$$

$$N4_{vrcjt} = 2 \cdot Q4_{vrcjt}/Q_{vj}^{max} \qquad \forall\, v \in V, r \in R, c \in C, j \in J, t \in T \qquad \text{(S39)}$$

$$N5_{vcwjt} = 2 \cdot (Q5_{vcwjt}/DPS)/Q_{vj}^{max} \qquad \forall\, v \in V, c \in C, w \in W, j \in J, t \in T \qquad \text{(S40)}$$

In addition, constraints are imposed on the maximum quantity of vaccines, Q^{max}, that can be transport per time period. In Equations S41 to S45, Q^{max} is fixed 10^{0}.

$$Q1_{vmwjt} \leq Q^{max} \qquad \forall\, v \in V, m \in M, w \in W, j \in J, t \in T \qquad \text{(S41)}$$

$Q2_{viwjt} \leq Q^{max}$ $\forall\, v \in V, w \in, r \in R, j \in J, t \in T$ (S42)

$Q3_{vwrjt} \leq Q^{max}$ $\forall\, v \in V, i \in I, w \in W, j \in J, t \in T$ (S43)

$Q4_{vrcjt} \leq Q^{max}$ $\forall\, v \in V, r \in R, c \in C, j \in J, t \in T$ (S44)

$Q5_{vcwjt} \leq Q^{max}$ $\forall\, v \in V, c \in c, w \in W, j \in J, t \in T$ (S45)

S3.3.6. SARS-CoV-2 and influenza vaccines demand

The demand of vaccines at administration points is fixed by the number of target individuals at a given region and/or number of individuals arriving at administration points. Equations S46 and S47 define the demand for influenza and SARS-CoV-2 vaccines respectively.

$D^S_{vct} \geq D^N_{vct}$ $\forall\, c \in C, t \in T, v \in FLU$ (S46)

$\sum_{v \in COV} D^S_{vct} \geq \sum_{v \in COV} D^N_{vct}$ $\forall\, c \in C, t \in T$ (S47)

where D^N_{vct} is the quantity of vaccines v needed at administration points, c, and time period t; while D^S_{vct}, is the quantity of vaccines supplied to vaccine administration points.

S3.3.7. Non-negativity constraints

In the supply chain model, all variables have non-negative values and this condition is enforced by Equation S48.

$All\ variables \geq 0$ (S48)

S3.4. Network configuration constraints

In the proposed mathematical formulation, binary variables E and X are used to indicate installation of facilities and establishment of connections/routes between supply chain entities. Here X denotes the existence of routes between adjacent supply chain entities, for example, $X1_{mw}$ denotes the route between manufacturing plants and warehouses. E denotes the existence of supply chain entities, for example $E1_m$, $E2_w$, $E3_r$, and $E5_c$ denote manufacturing plants, warehouses, regional stores, and administration points respectively. Equations S49 and S50 establish routes between manufacturing plant and warehouse only if the manufacturing plant and warehouse exist. The same concept applies to import locations to warehouses, warehouses to regional stores, regional stores to administration points, and administration points to warehouses, see Equations S51 to S53.

$X1_{mw} \leq E1_m$ and $X1_{mw} \leq E2_w$ $\forall\, m \in M, w \in W$ (S49)

$X2_{iw} \leq E2_w$ and $X2_{iw} \leq E5_r$ $\forall\, i \in I, w \in W$ (S50)

$X3_{wr} \leq E2_w$ and $X3_{wr} \leq E3_r$ $\quad \forall\, w \in W, r \in R$ (S51)

$X4_{rc} \leq E3_r$ and $X4_{rc} \leq E4_c$ $\quad \forall\, r \in R, c \in C$ (S52)

$X5_{cw} \leq E2_w$ and $X5_{cw} \leq E4_c$ $\quad \forall\, c \in C, w \in W$ (S53)

Vaccines are shipped between supply chain entities using three types of transportation modes: refrigerated van, refrigerated truck, and airfreight. The index j in Equations S54 to S58 represents the set of transportation modes.

$$Q1_{vmwjt} \leq X1_{mw} \cdot M^{Big} \quad \forall\, v \in V, m \in M, w \in W, j \in J, t \in T \tag{S54}$$

$$Q2_{viwjt} \leq X2_{iw} \cdot M^{Big} \quad \forall\, v \in V, i \in I, w \in W, j \in J, t \in T \tag{S55}$$

$$Q3_{vwrjt} \leq X3_{wr} \cdot M^{Big} \quad \forall\, v \in V, w \in W, r \in R, j \in J, t \in T \tag{S56}$$

$$Q4_{vrcjt} \leq X4_{rc} \cdot M^{Big} \quad \forall\, v \in V, r \in R, c \in C, j \in J, t \in T \tag{S57}$$

$$Q5_{vcwjt} \leq X5_{cw} \cdot M^{Big} \quad \forall\, v \in V, c \in C, w \in W, j \in J, t \in T \tag{S58}$$

In Equation S54, when a route is established between manufacturing plant *m* and warehouse *w*, vaccines are allowed to be shipped using a specific transport mode. $Q1_{vmwjt}$ denotes the quantity of vaccine *v* shipped from manufacturing plant *m* warehouse *w* using transportation mode *j* at time period *t*. Equations S55 to S58 define the shipment of vaccines/shippers between import location and warehouses, warehouses and regional stores, regional stores and administration points, administration points and warehouses respectively.
